# Huangqi injection in the treatment of chronic heart failure: A systematic review and meta-analysis

**DOI:** 10.1097/MD.0000000000008167

**Published:** 2017-09-29

**Authors:** Kaihuan Wang, Jiarui Wu, Xiaojiao Duan, Jiatao Wu, Dan Zhang, Xiaomeng Zhang, Bing Zhang

**Affiliations:** Department of Clinical Pharmacology of Traditional Chinese Medicine, School of Chinese Materia Medica, Beijing University of Chinese Medicine, Beijing, China.

**Keywords:** chronic heart failure, Huangqi injection, meta-analysis, systematic review

## Abstract

**Background:**

To evaluate the clinical effectiveness and safety of Huangqi injection (HI) in treating chronic heart failure (CHF) systematically.

**Methods:**

A literature search was conducted for retrieving randomized controlled trials (RCTs) on CHF treated by HI in the Cochrane Library, PubMed, Embase, China Biology Medicine disc, China National Knowledge Infrastructure Database, China Science and Technology Journal Database, Wanfang Database up to June, 6, 2017, and then the included RCTs were assessed by the Cochrane Risk of Bias Assessment Tool. The clinical total effective rate, left ventricular ejection fraction (LVEF), and others outcomes were analyzed by Review Manager 5.3 in random-effect model, the funnel plot were depicted as well. Meanwhile, the sensitivity analysis was carried out by STATA 12.0.

**Results:**

Sixteen RCTs involved 1864 patients were included. The result of HI group was more efficient in the clinical total effective rate (RR = 1.19, 95% confidence intervals (95% CI) [1.14–1.26], *P* < .00001). In addition, HI plus western medicine (WM) could improve LVEF (MD = 4.64, 95% CI [3.52–5.75], *P* < .00001), and others cardiac indexes. Meanwhile, a combination of HI and WM also can perfect 6 minutes walk test (6MWT). Three RCTs reported no serious adverse drug events/adverse drug reactions occurred.

**Conclusion:**

Compared with WM, a combination of HI and WM was more efficacious in improving the clinical total effective rate, and perfect patients’ condition, but more evidence-based medicine researches needed to support this study further.

## 1 Introduction

Chronic heart failure (CHF) is a cardiac diseases caused by heart overload, myocardial damage, and systolic dysfunction. If patients with cardiac diseases are deteriorated, their characteristics of CHF will be shown more distinctly.^[1–3]^ Besides, CHF remains a major reason leading to death all over the world,^[4–7]^ and there are almost 26 million people suffer from CHF throughout world.^[8]^

In terms of Traditional Chinese Medicine (TCM) theory, CHF pertains to “palpitation,” “edema,” primarily due to the deficiency of Qi and blood, phlegm–dampness, and blood stasis. The therapeutic principle is to strengthen body resistance and eliminate pathogen with TCM. Currently, the emphasis of treating CHF is to suppress myocardial remodeling, and perfect cardiac function. Thus, the combination of angiotensin converting enzyme inhibitor (ACEI), β-blocker, and aldosterone inhibitor becomes common for CHF; however, the problems such as poor compliance and low heart rate of patients may influence the desired effect.

Therefore, Chinese herbal injections (CHIs) are gradually applied to assisting in the treatment of CHF due to its rapid action, high bioavailability, and no digestive tract absorption, especially tonic CHIs. As a representative of tonic CHIs, Huangqi injection (HI) was approved by China Food and Drug Administration (CFDA) and has already achieved positive effect in treating CHF in clinical trials,^[9]^ while the conclusion of single trial was weak and its curative effect has not approved in clinical guideline. Hence, it is necessary to sort out and analyze relevant randomized controlled trials (RCTs) comprehensively. Based on existing clinical evidence, this study conducted a systematic review and meta-analysis to explore HI's effectiveness further and provide reference for adopting HI as well.

## 2 Methods

### 2.1 Search strategy

The following electronic databases were searched from its inception to June 6, 2017: the Cochrane Library, PubMed, Embase, China Biology Medicine disc, Chinese National Knowledge Infrastructure Database, China Science and Technology Journal Database, WanFang Database. The search method combining the medical subject headings (MeSH) term and free text word was used and changed into various forms with different database. Take the PubMed as the example, the strategy listed as follows:

#1 Heart Failure [MeSH Terms]

#2 Cardiac Failure[Title/Abstract] OR Heart Decompensation[Title/Abstract] OR Chronic heart failure[Title/Abstract] OR Myocardial Failure[Title/Abstract] OR Left-Sided Heart Failure[Title/Abstract] OR Left Sided Heart Failure[Title/Abstract] OR Right-Sided Heart Failure[Title/Abstract] OR Right Sided Heart Failure[Title/Abstract] OR Myocardial Failure[Title/Abstract] OR Congestive Heart Failure[Title/Abstract] OR Cardio-Renal Syndrome[Title/Abstract] OR Paroxysmal Dyspnea[Title/Abstract] OR Cardiac Edema[Title/Abstract]

#3 #1 OR #2

#4 huangqi OR Astragali radix

#5 #3 AND #4

### 2.2 Eligibility criteria

Eligible studies were: Types of studied: RCTs reported the effectiveness of HI in the treatment of CHF without limitation in languages. Types of patients: All involved patients should be diagnosed as CHF, the diagnostic standard was conformed to “Guidelines on the Diagnosis and Treatment of Heart Failure” conducted by The Chinese medical association cardiovascular epidemiology branch in 2014 or “Clinical Guideline of New Drugs for Traditional Chinese Medicine” released by CFDA in 2002.^[10,11]^ Included patients had no limitation in age, gender, race, national, and the severity of disease. Interventions: All patients received conventional western medicine (WM), for instance, cardiotonic, diuretic, ACEI, β-blocker, vasodilators, and so on. The experimental group received WM and HI, while the control group adopted the same WM. If patients suffered from others complication, clinician gave them corresponding therapies. Outcomes: Major outcomes contained clinical total effective rate and left ventricular ejection fraction (LVEF). Besides, the incidence of left ventricular end-diastolic volume (LVEDV), left ventricular end-systolic volume (LVESV), stroke cardiac output (SV), cardiac output (CO), 6 minutes walk test (6MWT), and safety situation (adverse drug reactions (ADRs)/adverse drug events (ADEs)) were also evaluated and deemed as secondary outcomes. The clinical total effective rate calculated by this formula: (number of remarkable recovery patients + number of basic recovery patients)/total number of patients × 100%. According to cardiac function classification standard issued by New York Heart Association in the United States (NYHA), clinical symptoms disappeared and cardiac function perfected 2 level belonged to the class of remarkable recovery patients, clinical symptoms eased and cardiac function improved 1 level was classified into the part of basic recovery, clinical symptoms and cardiac function were unchanged or worsened pertained to grade of deterioration. The studies were excluded if WM contained rehabilitation therapy and naturopathy. The experimental group and the control group's intervention included others TCM treatments except CHIs, for instance acupuncture, moxibustion, and Chinese patent medicine. The data of outcomes were incomplete. The literature cannot obtain full text. If 2 literatures had the same data, we utilized the data of the literature with the larger sample and relative complete information.

### 2.3 Data extraction and quality assessment

Two reviewers carried out literature filtration complying with predesigned eligible criteria, and extracted the included RCTs’ information in Microsoft Excel 2010 (Microsoft Crop, Redmond, WA). The necessary information comprised these aspects: The essential information of included RCTs: the first author, public date. The characteristics of patients: the number of the experimental group and the control group, gender proportion, age. The details of intervention: specific therapies and there dosages, treatment courses. The data of outcomes. The key elements of risk assessment: randomization, blinding, and handing of dropouts. All literatures were managed by NoteExpress (Wuhan University Library, Wuhan, China).

Methodological quality assessment of each RCT was conducted by the Cochrane Risk of Bias Assessment Tool,^[12]^ which contained 7 aspects: sequence generation (selection bias), allocation concealment (selection bias), blinding of patients and personnel (performance bias), blinding of outcome assessment (detection bias), incomplete outcome data (attrition bias), selective outcome reporting (reporting bias), and other sources of bias. Each aspect was classified into 3 levels: low risk, unclear risk, and high risk. If the RCTs described a correct random generation, implemented blinding and reported complete measure outcomes, the RCTs belonged to low risk. On the contrary, the trails pertained to high risk. Besides, the RCTs were deemed as unclear risk provided that the literature did not provide enough information for judgments.^[12]^ Two reviewers accessed the quality of RCTs separately, and any inconsistency between 2 reviewers was resolved by consulting the third reviewer.

Based on collecting clinical experiments’ data, this study would not leak out patients’ information. Thus, it is unnecessary to conduct the ethical approval for this study.

### 2.4 Statistical analysis

Review Manager 5.3 (Cochrane Collaboration, Oxford, UK) was utilized to synthesize and analyze data. As for dichotomous outcomes, relative risk (RR) was applied to count data, whereas mean difference (MD) was used to evaluate continuous variable, 95% confidence intervals (95% CIs) was presented as well in order to indicate the range of results. Heterogeneity was given as Chi^2^, *I*^2^, and tau^2^.^[13]^ And the random-effect model in inverse variance method was applied into meta-analysis.^[14]^ Meanwhile, several methods were used to evaluate publication bias, visual inspection was demonstrated by funnel plot. And Egger test and Begg test were also adopted. In addition, the sensitivity analysis was conducted in clinical total effective rate so as to test the stability of results. The sensitivity analysis was done by excluding one of the RCT at a time and then reconducting meta-analysis. Egger test, Begg test, and sensitivity analysis were estimated by STATA 12.0^[15]^ (StataCorp LP, College Station, TX).

## 3 Results

### 3.1 Study characteristics

**Figure 1:**
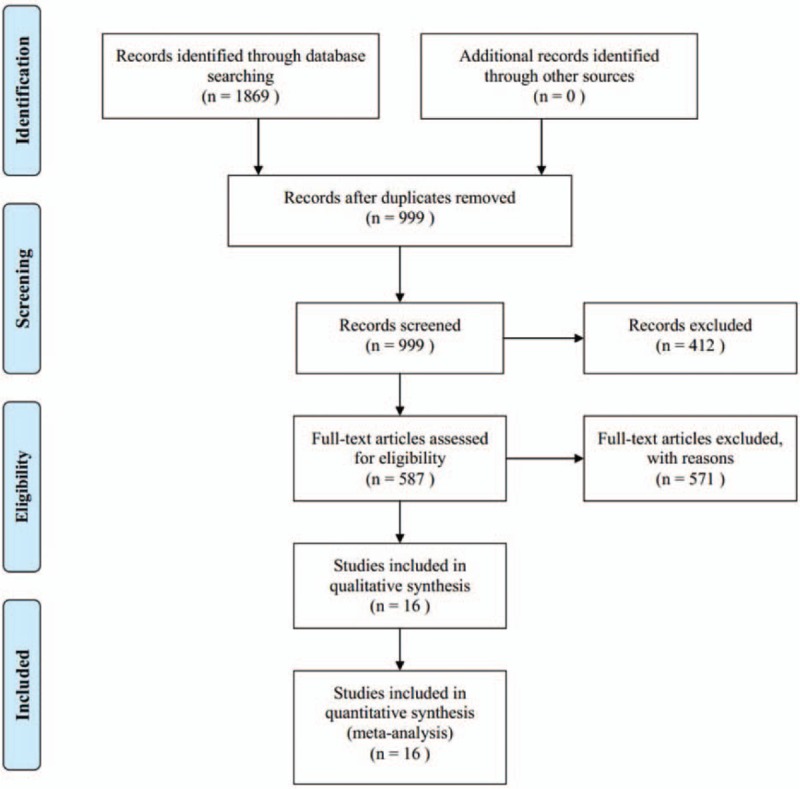
Flow chart of literature search.

Our search identified 1869 literatures initially in total through the 7 databases. NoteExpress checked duplications automatically, the 999 literatures remained. After reading the titles and abstracts by 2 reviewers respectively, reviews, irrelative literatures, and animals experiments were excluded. Then, 577 literatures remained, of which 561 were excluded by reading the full text due to the following reasons: not diagnosed as CHF (119 literatures), not complied with the intervention of inclusion criteria (287 literatures), not referred to the diagnostic standard or therapeutic criterion (132 literatures), not performed in 2 groups (10 literatures), cannot contain the full text (6 literatures), individual cases (1 literature), the same literature (6 literatures). The final 16 RCTs were included in this study, all of them published in Chinese from 2003 to 2017. The flow diagram about the filtration is depicted in Fig. 1.

Sixteen RCTs involved 1273 patients, among which 656 patients in experimental group and 617 patients in control group. All patients were diagnosed as CHF and conformed to eligible criteria, male patients accounted for 59.1% (752/1273) and the middle-age and elderly patients were majority. In these 16 RCTs, the sample size ranged from 43 to 159. As for intervention, the control groups were digoxin, furosemide, captopril, metoprolol, isosorbide dinitrate, and other WM treatments, the experimental group injected HI plus the same WM. All patients received treatment once a day via mainline, and the period of treatment was within 30 days The period was 14 to 15 days and the dosage of HI was 30 to 40 mL in most RCTs. Characteristics of eligible RCTs are summarized in Table 1.

**Table 1:**
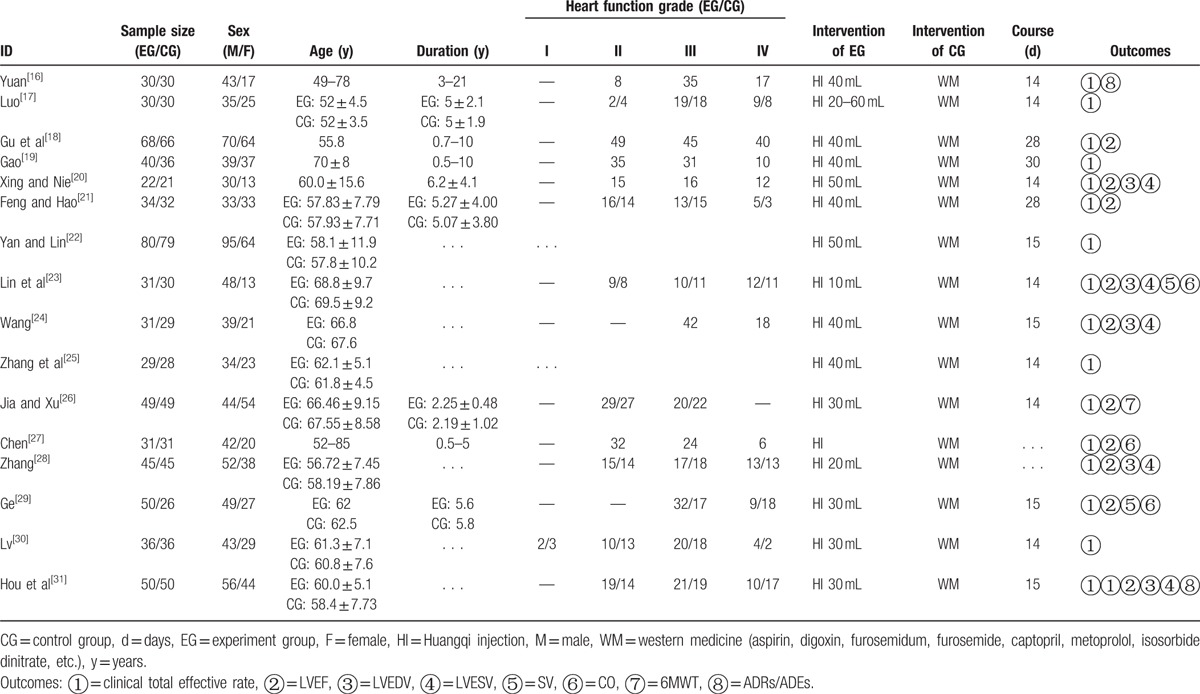
Study characteristics.

**Figure 2:**
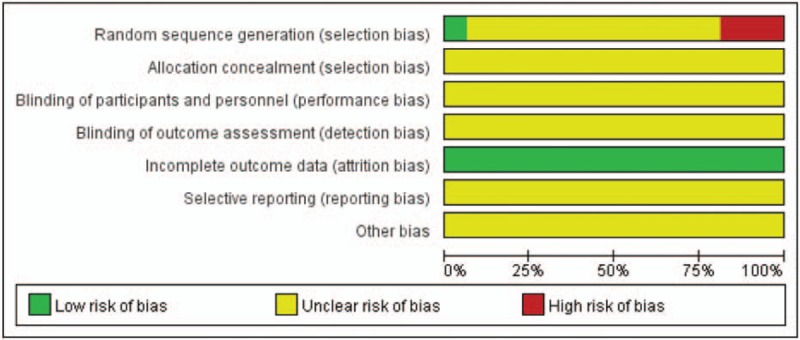
Risk of bias summary.

In 16 RCTs, 1 RCT used random number table to generate the group,^[28]^ and 3 RCTs generated groups by the order of hospitalization.^[29–31]^ The others did not provide the details of randomization in the literatures.^[16–27]^ Thus, the bias resulted from random sequence generation of them was evaluated as “low,” “high,” and “unclear.” The information on allocation concealment, blinding was not observed in the literatures. Hence this study evaluated the bias of allocation concealment bias, performance bias, and detection bias as “unclear.” Besides, none of the included RCTs assessed had incomplete data, so the attrition bias was appraised as “low.” As for the part of reporting bias and other bias, 16 RCTs did not provide relevant contents about selective reporting and mention any factors leading to high risk. Therefore, these 2 items were evaluated as “unclear.” The quality of the included RCTs is demonstrated in Fig. 2.

### 3.2 Outcomes

#### 3.2.1 Clinical total effective rate

**Figure 3:**
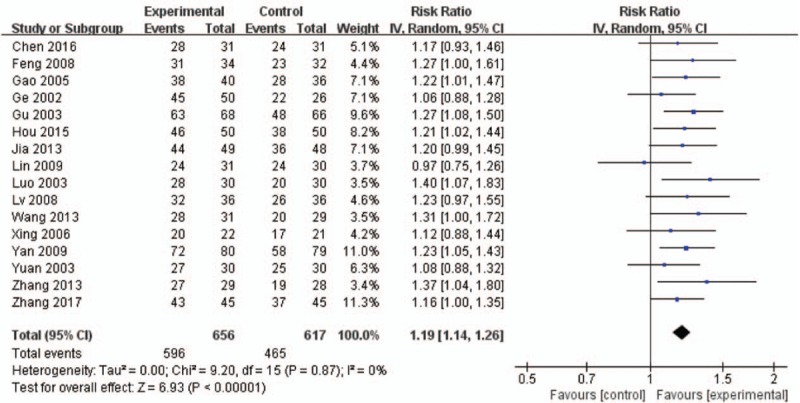
Forest plot of the clinical total effective rate.

All of the 16 RCTs tested the clinical total effective rate. Pooled results showed an improvement in favor of the experimental group on clinical total effective rate (RR = 1.19, 95% CI: 1.14–1.26, *P* < .00001, *I*^2^ = 0%, tau^2^ = 0.00, Fig. 3).

#### 3.2.2 Sensitivity analysis and publication bias

**Figure 4:**
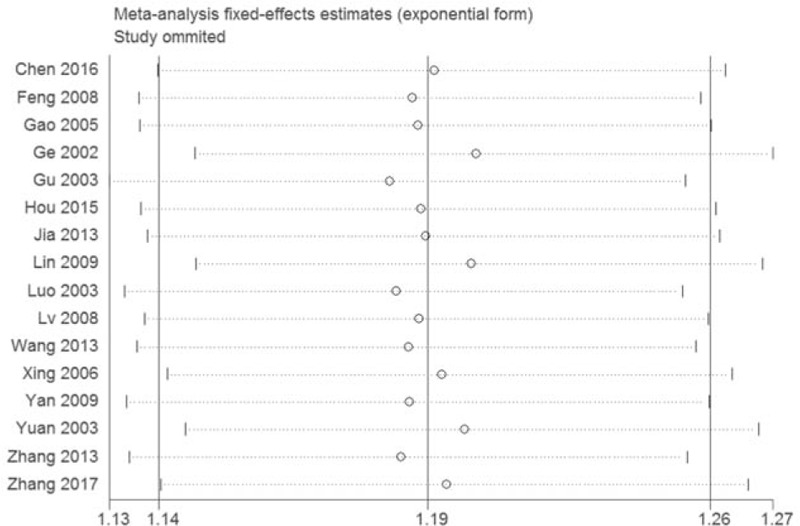
Sensitivity analysis of the clinical total effective rate.

This study conducted a sensitivity analysis focusing on the clinical total effective rate to verify its stability, which has done by eliminating one item at a time and then reanalysis. The results of sensitivity is shown in Fig. 4, the result of clinical total effective rate did not appear a qualitative transform which meant this outcome had a good stability.

**Figure 5:**
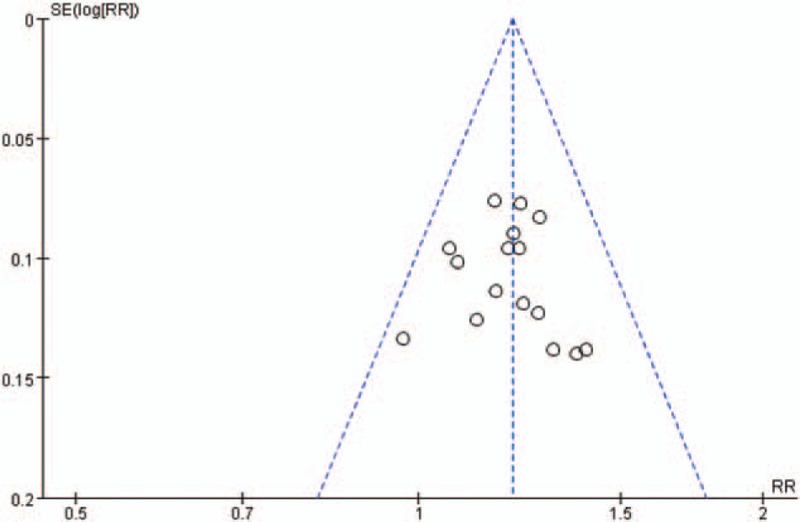
Funnel plot of the clinical total effective rate.

A funnel plot on publication bias for clinical total effective rate is displayed in Fig. 5, and included RCTs distributed evenly. Moreover, the result of Egger test (*t* = 0.38, *P* = .707 > .05) and Begg test (*z* = 0.95, *P* = .344 > .05) indicated no evidence of significant publication bias.

#### 3.2.3 LVEF

**Figure 6:**
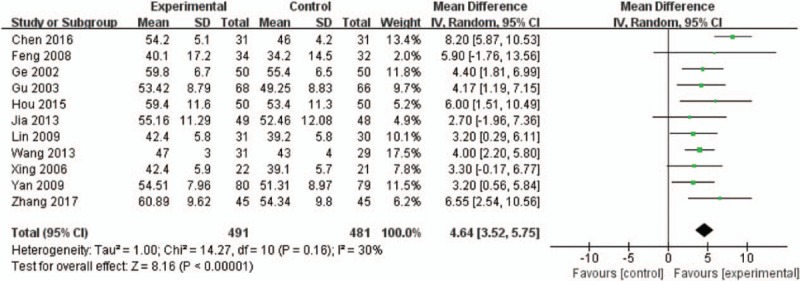
Forest plot of LVEF.

LVEF was compared with in 10 RCTs. If the MD value of meta-analysis was higher, the improvement of LVEF was relative superior. The aggregated data indicated that HI plus WM had a better impact on increasing LVEF than WM (MD = 4.64, 95% CI: 3.52–5.75, *P* < .00001, *I*^2^ = 30%, tau^2^ = 1.00, Fig. 6).

#### 3.2.4 Secondary outcomes

Detailed data presented in Table 2.

(1) LVEDV: 4 RCTs examined the LVEDV.^[20,23,28,31]^ The overall results demonstrated that a combination of HI and WM was excelled at lowering LVEDV than WM.
(2) LVESV: 4 RCTs reported the LVESV.^[20,23,28,31]^ Pooled results signified that HI plus WM exhibited a perforable effect on decreasing LVESV more effectively compared with WM.
(3) SV: 3 RCTs investigated SV.^[23,24,29]^ The results manifested that the conjunctive use of HI and WM can perform a good effect on enhancing SV.
(4) CO: 3 RCTs identified SV.^[23,27,29]^ The results indicated that HI plus WM was more efficient in promoting CO in comparing with WM.
(5) 6MWT: 1 RCT tested 6MWT.^[26]^ Thus, this study made a qualitative description for it. After treatment, the result of the experimental group was 386 ± 52.06 m, whereas the control group was 326.05 ± 32.14 m, the difference between 2 groups was significant as well.

**Table 2:**
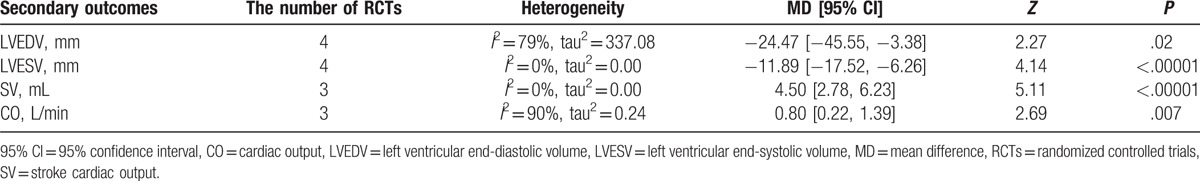
Detailed data of secondary outcomes.

#### 3.2.5 Safety

Among 16 RCTs, a total of 3 RCTs mentioned that there were no obviously ADRs/ADEs.^[16,24,31]^ The other RCTs did not reported about ADRs/ADEs.

## 4 Discussion

HI is a common CHI that manufactured by the extractive Huangqi (*Astragali radix*) under the guideline of TCM, and its effective constituents are astragaloside, astragalus polysaccharide, and so forth.^[32]^ Huangqi is well known for its characters in removing blood stasis without injuring righteousness and widely used as Qi-tonifying herbal medicine.^[33,34]^ The results of pharmacological experiments manifested that Huangqi can enhance myocardial contractility and myocardial cell excitation–contraction coupling. Then it can generate significant cardiotonic effect, whose functions are similar to the positive inotropic effect of digitalis. Beyond that, Huangqi owns a capacity not only on increasing SOD, but also on cleaning oxygen free radical to prevent them from damaging myocardial cell. In addition, Huangqi is capable of decreasing cardiac pressure load and volume load via vasodilation, which may result in slowing heart rate, reducing release kallikrein from central nervous system and renin angiotensin aldosterone system.^[35–38]^ Theoretically, HI is a better option for treating CHF.

The results of this study suggested that, in terms of CHF patients, an integration of HI and WM can make a remarkable influence than WM in improving clinical effective rate and LVEF, lessening LVEDV and LVESV, perfecting SV and CO as well. Beyond that, HI plus WM also had a notable performance on increasing 6MWT. As for its safety, 3 RCTs reported that there were no serious ADRs/ADEs occurred in the treatment. While due to the small quantity, it cannot draw a robust conclusion that HI had a well safety for treating CHF. Besides, the dosage of included RCTs was above the ruled usage (the specification stipulate 10–20 mL once a day), which was irrational and may engender ADRs/ADEs. In addition, elderly patients were in large proportion among included patients who may likely drive the drug build-up and then arised accumulation of drug. Therefore, clinical should pay more attention on the usage.^[39]^

There were 3 systemic reviews focused on the efficacy of HI for treating CHF respectively published in 2008, 2009, 2011.^[40–42]^ One of them contained 62 RCTs and quasi-RCTs, the rest literatures contained 10 RCTs and 11 RCTs. All of them compared the effectiveness between HI plus WM with WM, one of them contrasted HI and nitroglycerin as well. Apart that, the clinical total effective rate and LVEF were deemed as primary outcomes. By contrast, this study owned the following advantages: firstly, we updated the search date to June 6, 2017 and made a relative comprehensive retrieval in the Cochrane Library, PubMed, Embase, China Biology Medicine disc, Chinese National Knowledge Infrastructure Database, China Science and Technology Journal Database, WanFang Database by combining MeSH term with free text word. Secondly, in order to lower clinical heterogeneity, we formulated strict eligible criteria. In order to ensure the identical base line, all included patients diagnosed as CHF in specific criteria and evaluated the effectiveness in congruent standard, and received the same interventions as well. Thirdly, besides the clinical total effective rate and LVEF, this study also set LVEDV, LVESV, SV, CO as outcomes aimed to indicate cardiac situation. Meanwhile, because 1 RCT reported 6MWT, we utilized a descriptive method in narrating its results to reflect the recovery of patients.

## 5 Limitations

This study was not without limitations. Firstly, included RCTs’ quality was general, and most items were assessed as unclear risk, which may influence the validity of overall findings and overestimate the effectiveness of HI to some degree. Meanwhile, though the Egger test and Begg test manifested that there was no potential publication bias, the deficiency of the funnel plot's top and bottom may also indicated that this study lack the RCTs with very small or large sample. Besides, the original reports of RCTs did not report the contents of intention-to-treat analysis, the missing of it may affect the strength of this evidence. Secondly, all of the RCTs did not conduct follow-up visit after treatment. Thus, whether there was any significant difference between 2 groups in the long-term curative effect like recurrence rate and mortality cannot figure out. Apart that, it is 6MWT that can reflect patients’ situation directly, while this outcome was examined in 1 RCT. Thirdly, this study cannot draw a tangible conclusion on safety due to inadequate ADRs/ADEs information. On account of foregoing shortcoming, we raised several suggestions towards RCTs on HI. Firstly, in order to ensure the transparency of RCTs’ process, it is necessary that RCTs should be registered in advance and carried out accord with Consort standard.^[43,44]^ Meanwhile, randomization, allocation concealment, and blinding ought to perform as possible. Secondly, clinicians had better chose outcomes that associate closely with patients’ feelings and the long-term effectiveness. Thirdly, the clinicians ought to strengthen monitoring of ADRs/ADEs situation while they concern the effectiveness.

## 6 Conclusion

To sum up, this study showed that a combination of HI and WM was beneficial for clinical total effective rate, LVEF, LVEDV, LVESV, and others cardiac indexes. Besides, it also can promote 6MWT. However, due to limitations of included RCTs, high quality RCTs with scientific rigor and strict implement are needed to testify HI's effectiveness further.
